# Impact of Endoscopic Ultrasonography on ^18^F-FDG-PET/CT Upfront Towards Patient Specific Esophageal Cancer Treatment

**DOI:** 10.1245/s10434-017-5835-1

**Published:** 2017-03-16

**Authors:** J. B. Hulshoff, V. E. M. Mul, H. E. M. de Boer, W. Noordzij, T. Korteweg, H. M. van Dullemen, W. B. Nagengast, V. Oppedijk, J. P. E. N. Pierie, John Th. M. Plukker

**Affiliations:** 1 Department of Surgical Oncology, University Medical Center Groningen, University of Groningen, Groningen, The Netherlands; 2 Department of Radiotherapy, University Medical Center Groningen, University of Groningen, Groningen, The Netherlands; 3 Department of Nuclear Medicine and Molecular Imaging, University Medical Center Groningen, University of Groningen, Groningen, The Netherlands; 4 Department of Radiology, University Medical Center Groningen, University of Groningen, Groningen, The Netherlands; 5 Department of Gastroenterology and Hepatology, University Medical Center Groningen, University of Groningen, Groningen, The Netherlands; 6 Radiotherapeutic Institution Friesland, Leeuwarden, The Netherlands; 7 Postgraduate School of Medicine, Groningen, The Netherlands; 8 Surgery Department, Medical Center Leeuwarden, Leeuwarden, The Netherlands

## Abstract

**Introduction:**

In patients with potentially resectable esophageal cancer (EC), the value of endoscopic ultrasonography (EUS) after fluorine-18 labeled fluorodeoxyglucose positron emission tomography with computed tomography (^18^F-FDG-PET/CT) is questionable. Retrospectively, we assessed the impact of EUS after PET/CT on the given treatment in EC patients.

**Methods:**

During the period 2009–2015, 318 EC patients were staged as T1-4aN0-3M0 with hybrid ^18^F-FDG-PET/CT or ^18^F-FDG-PET with CT and EUS if applicable in a nonspecific order. We determined the impact of EUS on the given treatment in 279 patients who also were staged with EUS. EUS had clinical consequences if it changed curability, extent of radiation fields or lymph node resection (AJCC stations 2–5), and when the performed fine-needle aspiration (FNA) provided conclusive information of suspicious lymph node.

**Results:**

EUS had an impact in 80 (28.7%) patients; it changed the radiation field in 63 (22.6%), curability in 5 (1.8%), lymphadenectomy in 48 (17.2%), and FNA was additional in 21 (7.5%). In patients treated with nCRT (*n* = 194), EUS influenced treatment in 53 (27.3%) patients; in 38 (19.6%) the radiation field changed, in 3 (1.5%) the curability, in 35 (18.0%) the lymphadenectomy, and in 17 (8.8%) FNA was additional. EUS influenced both the extent of radiation field and nodal resection in 31 (16.0%) nCRT patients.

**Conclusions:**

EUS had an impact on the given treatment in approximately 29%. In most patients, the magnitude of EUS found expression in the extent of radiotherapy target volume delineation to upper/high mediastinal lymph nodes.

Accurate staging is essential for oncological outcome in patients with potentially curable esophageal cancer for determining the optimal treatment approach and adequate target volume delineation (gross tumor volume of primary tumor and malignant nodes and hence clinical target volume) when radiotherapy is indicated. Fluorine-18 labeled fluorodeoxyglucose positron emission tomography with computed tomography (^18^F-FDG-PET/CT) is currently the best method to detect distant metastases, whereas endoscopic ultrasonography (EUS) with or without fine-needle aspiration (FNA) is the method of choice to determine the extent and depth-growth of the primary tumor (T-stage) and detection of lymph node metastases (N-stage).^1^–^3^

Contradictory results have been reported regarding the additional role of EUS after ^18^F-FDG-PET, ^18^F-FDG-PET/CT or CT alone.^4^–^8^ Mortensen et al. found that EUS influenced treatment decisions in 34% of the EC patients after CT alone, whereas Findlay et al. found that the risks outweighed the potential benefit of EUS in patients with T2-4a disease on CT.^4^,^5^ Some studies showed upfront ^18^F-FDG-PET followed by EUS as the best predictor of curative resectability and the most cost-effective staging sequence.^7^,^8^ However, most studies determined the influence of EUS with questionnaires completed by involved medical specialists and not with the clinical impact or diagnostic position of EUS after ^18^F-FDG-PET/CT.^4^,^6^ Moreover, these studies did not assess the magnitude of EUS on radiotherapy target volume delineation; nowadays most patients receive radiotherapy in a combined curative treatment approach.

In addition, EUS has several limitations, including the invasive character of the procedure with risk of aspiration, perforation, and bleeding, whereas tumor stenosis, which occurs between 20 and 36% of the patients, limits its clinical usage.^3^,^9^–^11^ Precluding unnecessary EUS is therefore patient-friendly in case of a strong suspicion of distant metastases or in case of localized disease on ^18^F-FDG-PET/CT and reduces the risks of complications and eventually costs.

With ^18^F-FDG-PET/CT upfront staging sequence, unnecessary primary endoscopic procedures may be prevented, by ^18^F-FDG-PET/CT guiding of concurrent EUS with fine needle aspiration (FNA) of pathological (non)regional lymph nodes.

The purpose of this study was to determine the clinical impact of EUS after upfront ^18^F-FDG-PET/CT staging on the given treatment, by determining if EUS had changed radiotherapy target volume delineation, curability, extent of lymph node resection, and whether FNA was of additive value. We also investigated the additional value of EUS on nodal up/downstaging, on the number of lymph nodes suspected for metastasis, and station-specific nodal (N) status.

## Patients and Methods

A total of 318 patients were included in this retrospective study, which was performed according to the guidelines of the local Ethical Boards and national rules for retrospective studies. All esophageal cancer patients (*n* = 388) scheduled for an in onset curative intended treatment (T1-4aN0-3M0), diagnosed at the University Medical Center Groningen (UMCG) or Medical Center Leeuwarden between 2009 and 2015, were eligible for inclusion. All consecutive patients had a pathologically proven adeno- or squamous cell carcinoma, located in the mid or distal esophagus or at the gastroesophageal junction. Excluded were patients treated with endoscopic mucosal resection before (*n* = 7) or after (*n* = 3) staging, without ^18^F-FDG-PET or ^18^F-FDG-PET/CT scanning (*n* = 17), without a visible tumor on ^18^F-FDG-PET/CT (*n* = 15), or with missing staging data (*n* = 28).

### Methods

First, we determined the number of patients who had a complete or incomplete EUS and those without EUS. To answer the primary research question, all patients with a complete or incomplete EUS were selected. We then assessed if EUS had an impact on the given treatment. Compared with the noninvasive ^18^F-FDG-PET/CT, EUS had influenced the given treatment if: EUS changed the extent of radiation target volumes (smaller, larger, or both), changed the curability (from incurable based on ^18^F-FDG-PET/CT to potentially curable), influenced the extent of lymph node dissection (i.e., changed suspicion for lymph node metastasis at AJCC lymph node stations 2–5), or/and when the performed fine-needle aspiration (FNA) provided conclusive information of suspicious lymph node.^12^ Thereafter, we assessed the clinical impact of EUS on the given treatment in the total patient group (all treatments with curative intent), followed by the neoadjuvant chemoradiotherapy (nCRT), the surgery-alone, and a combined (chemoradiotherapy and definitive radiotherapy (dCRT/dRT) group.

The researchers (JBH & VEMM) assessed the radiotherapy tumor volume (TV) delineations and determined if the EUS findings had changed the radiotherapy target volumes. EUS enlarged the radiotherapy target volume delineation if EUS-FNA had identified new lymph node suspicious for metastases that were located outside of the clinical target volume (CTV: treatment section) based on the ^18^F-FDG-PET/CT. Lymph nodes were scored by size, shape, echoic pattern, and sharpness of the border. EUS decreased the radiotherapy target volume delineation if initially suspected lymph nodes on the ^18^F-FDG-PET/CT, located outside of the CTV, were reassessed as negative based on EUS findings and/or confirmed cytologically as FNA-negative. Secondary outcomes included the number of patients with an altered N-stage, with a difference in the number of lymph nodes suspected for metastases, and changes in the localization of suspected lymph nodes.

### Staging

Patients were staged according to the 7th TNM classification of the American Joint Committee on Cancer (AJCC) with diagnostic gastroscopy and biopsy, a diagnostic CT thorax/abdomen, ^18^F-FDG-PET or ^18^F-FDG-PET/CT, and EUS if possible.^13^ Depending on the tumor-specific situation, EUS was performed with either a high-frequency (12–20 MHz) linear or radial probes. Esophageal dilatation was not performed routinely in patients with stenosis. The results of all staging methods were discussed in a multidisciplinary tumor board. Abnormalities and non-regional lesions relatively suspect for being a distant metastasis were either proven pathologically under imaging guided biopsies or assessed with additional imaging techniques i.e., magnetic resonance imaging (MRI) or endobronchial ultrasound/FNA.

### Treatment

nCRT followed by surgery consisted of carboplatin and paclitaxel according to the CROSS regimen combined with radiotherapy (41.4 Gy/23 fractions).^14^ dCRT consisted of either carboplatin/paclitaxel or cisplatin and fluorouracil (Cis-5FU) combined with radiotherapy (50.4 Gy).^15^ dRT commonly consisted of 60 Gy in 30 fractions.

In all patients who received external beam radiotherapy, the gross tumor volume (GTV) was delineated on a planning CT scan, using all additional staging information. The GTV contained both the primary tumor and adjacent lymph nodes. The clinical target volume (CTV) is a margin of 3–3.5 cm in cranial and caudal direction and 1–2 cm transversally. The CTV was adjusted to normal tissue. Suspicious lymph nodes located outside the CTV were radiated separately with 1-cm margin.

Surgery consisted of either a transthoracic, transhiatal, or minimally invasive radical esophagectomy with a two-field lymph node dissection and was normally performed 6–10 weeks after the end of nCRT.

### Statistical Analysis

All data were displayed as numbers (percentages). Normally distributed data were displayed as mean (standard deviation). Nonnormally distributed data were displayed as median [interquartile range (IQR)]. Differences in categorical variables were assessed using logistic regression, Chi square test, or likelihood ratio. All data were analyzed using SPSS statistical software, version 22 (SPSS Inc., Chicago, IL).

## Results

Table 1 displays the patients’ characteristics, tumor-related, and treatment-related data of the 318 included patients. Of these patients, 250 (78.6%) had an adenocarcinoma and 68 (21.4%) had a squamous cell carcinoma. The localization of the primary tumor was generally in the distal esophagus (*n* = 221: 69.5%), followed by the gastroesophageal junction (*n* = 52: 16.4%), and mid esophagus (*n* = 45: 14.2%). Most patients were treated with curative intent with surgery after nCRT (*n* = 212: 66.7%), followed by surgery-alone (*n* = 50: 15.7%), dCRT (*n* = 29: 9.1%), and dRT (*n* = 27: 8.5%).

**Table 1:** Patient, tumor, and treatment characteristics

	All patients (*n* = 318), *n* (%)	Patients with a complete EUS (*n* = 279), *n* (%)
Male	248 (78.0%)	216 (77.4%)
Age (year); median (IQR)	65 (59–70)	65 (60–70)
Histology		
Adenocarcinoma	250 (78.6%)	225 (80.6%)
Squamous cell carcinoma	68 (21.4%)	54 (19.4%)
Tumor location		
Middle esophagus	45 (14.2%)	34 (12.2%)
Distal esophagus	221 (69.5%)	198 (71.0%)
GEJ	52 (16.4%)	47 (16.8%)
Tumor length (cm); median (IQR)	5.0 (3.0-7.0)	5.0 (3.0-6.0)
cT-stage		
T1	11 (3.5%)	10 (3.6%)
T2	41 (12.9%)	39 (14.0%)
T3	243 (76.4%)	218 (78.1%)
T4a	23 (7.2%)	12 (4.3%)
cN-stage		
N0	85 (26.7%)	70 (25.1%)
N1	117 (36.8%)	100 (35.8%)
N2	99 (31.1%)	93 (33.3%)
N3	15 (4.7%)	15 (5.4%)
Missing	2 (0.6%)	1 (0.4%)
Treatment		
Surgery-alone	50 (15.7%)	45 (16.1%)
nCRT	212 (66.7%)	194 (69.5%)
dCRT	29 (9.1%)	21 (7.5%)
dRT	27 (8.5%)	19 (6.8%)

*IQR* interquartile range, *GEJ* gastroesophageal junction, *cT*-*stage* clinical T-stage, *cN*-*stage* clinical N-stage, *nCRT* neoadjuvant chemoradiotherapy, *dCRT* definitive chemoradiotherapy, *dRT* definitive radiotherapy

Table 2 displays the endoscopic ultrasonography-related data. EUS was not performed in 39 (12.3%) patients because of stenosis (*n* = 30/9.4%), patient related reasons (*n* = 4), and for unknown reasons (*n* = 5). In total, EUS could be performed in 279 patients: 200 (62.9%) had a complete and 79 (24.8%) an incomplete EUS, because of stenosis (*n* = 78/24.5%) and patients related reason (*n* = 1).

**Table 2:** Endoscopic ultrasonography-related data

	All patients (*n* = 318), *n* (%)
EUS successful	200 (62.9%)
EUS incomplete	79 (24.8%)
Stenosis	78 (24.5%)
Patient-related	1 (0.3%)
EUS not performed	39 (12.3%)
Stenosis	30 (9.4%)
Patient related	4 (1.3%)
On indication no EUS	1 (0.3%)
Unknown	4 (1.3%)

### Influence of EUS on the Given Treatment

Table 3 displays the additional information that EUS provided compared with upfront ^18^F-FDG-PET/CT. EUS had influenced the primary treatment in 80 (28.7%) patients. In most patients (*n* = 63: 22.6%), EUS influenced the extent of radiation target volumes: by increasing the radiotherapy target volume delineation in 45 (16.1%) patients, decreasing these delineation in 17 (6.1%), and both extending and decreasing the target volume in 1 (0.4%) patient. EUS had changed the curability (incurable to potentially curable) in 5 (1.8%) patients, influenced the extent of LN resection (at node level AJCC Lymph node stations 2–5) in 48 (17.2%), and affected treatment decision-making with FNA in 21 patients (7.5%).

**Table 3:** Influence of EUS compared to ^18^F-FDG-PET/CT

	All patients (*n* = 279), *n* (%)	nCRT (*n* = 194), *n* (%)	Surgery-alone (*n* = 45), *n* (%)	dCRT & dRT (*n* = 40), *n* (%)
EUS influenced the treatment	80 (28.7%)	53 (27.3%)	13 (28.9%)	14 (35.0%)
Radiotherapy fields	63 (22.6%)	52 (26.8%)	n.a.	11 (27.5%)
Larger RT field	45 (16.1%)	38 (19.6%)	n.a.	7 (17.5%)
Smaller RT field	17 (6.1%)	13 (6.7%)	n.a.	4 (10.0%)
Smaller and larger	1 (0.4%)	1 (0.5%)	n.a.	0 (0.0%)
Incurable → curable	5 (1.8%)	3 (1.5%)	0 (0.0%)	2 (5.0%)
Influence on LN resection	48 (17.2%)	35 (18.0%)	13 (28.9%)	n.a.
Positive on EUS	29 (10.4%)	20 (10.3%)	9 (20.0%)	n.a.
Negative on EUS	19 (6.8%)	15 (7.7%)	4 (8.9%)	n.a.
FNA additional	21 (7.5%)	17 (8.8%)	3 (6.7%)	1 (2.5%)
Fine-needle aspiration				
Positive	22 (7.9%)	14 (7.2%)	1 (2.2%)	7 (17.5%)
Negative	31 (11.1%)	27 (13.9%)	4 (8.9%)	0 (0.0%)
Inconclusive	5 (1.8%)	2 (1.0%)	2 (4.4%)	1 (2.5%)
N up/down staging				
up staging	107 (38.4%)	87 (44.8%)	12 (26.7%)	8 (20.0%)
down staging	31 (11.1%)	15 (7.7%)	9 (20.0%)	7 (17.5%)
More LN found with EUS	150 (53.8%)	113 (58.2%)	18 (40.0%)	19 (47.5%)
Less LN found with EUS	32 (11.5%)	16 (8.2%)	9 (20.0%)	7 (17.5%)
Other LN localization	77 (27.6%)	52 (26.8%)	11 (24.4%)	14 (35.0%)
Additional staging techniques				
Bronchoscopy	8 (2.9%)	2 (1.0%)	0 (0.0%)	6 (15.0%)
EBUS	6 (2.2%)	4 (2.1%)	1 (2.2%)	1 (2.5%)
Ultrasound liver	11 (3.9%)	6 (3.1%)	4 (8.9%)	1 (2.5%)
Ultrasound neck	21 (7.5%)	16 (8.2%)	2 (4.4%)	3 (7.5%)
MRI liver	1 (0.4%)	1 (0.5%)	0 (0.0%)	0 (0.0%)
CT liver	1 (0.4%)	0 (0.0%)	0 (0.0%)	1 (2.5%)
CT thorax	2 (0.7%)	2 (1.0%)	0 (0.0%)	0 (0.0%)
CT brain	1 (0.4%)	1 (0.5%)	0 (0.0%)	0 (0.0%)
Mediastinoscopy	1 (0.4%)	0 (0.0%)	1 (2.2%)	0 (0.0%)
Number of EUS				
1	272 (97.5%)	191 (98.5%)	45 (100%)	36 (90.0%)
2	7 (2.5%)	3 (1.5%)	0 (0.0%)	4 (10.0%)

*nCRT* neoadjuvant chemoradiotherapy, *dCRT* definitive chemoradiotherapy, *dRT* definitive radiotherapy, *EUS* endoscopic ultrasonography, *RT* radiotherapy, *LN* lymph node, *FNA* fine-needle aspiration, *EBUS* endobronchial ultrasound, *n.a.* not applicable

Thereafter, we found that histologic tumor type did not influence the effect of EUS on the given treatment (*P* = 0.614). The location of the primary tumor also influenced the effect of EUS on the given treatment (*P* = 0.043): midesophageal-located cancers interfered with the treatment in 16 (47.1%), distal EC in 50 (25.3%), and gastroesophageal junction tumors in 14 (29.8%) patients.

In the nCRT group (*n* = 194), EUS changed the proposed treatment based on upfront ^18^F-FDG-PET-CT in 53 (27.3%). In 52 (26.8%) patients, it influenced the radiation target volumes, in 3 (1.5%) the curability, in 35 (18.0%) the extent of lymph node dissection, and in 17 (8.8%) FNA added valuable information. Of all patients, in 31 (16.0%) EUS affected the extent of both, the radiation target volumes and the lymph node dissection, usually at AJCC lymph node 2–5, implicating EUS to be valuable in identifying mediastinal lymph node metastases, especially these upper/high lymph nodes.

EUS influenced the treatment in 13 (28.9%) surgery-alone treated patients (*n* = 45), all by extending the lymph node dissection: EUS identified mediastinal/high lymph node metastasis in 9 (20.0%) patients with ^18^F-FDG-PET/CT positive lymph nodes, whereas in 4 (8.9%) other patients the ^18^F-FDG-PET/CT positive lymph nodes turned out to be negative on EUS. In 3 (6.7%) of these 13 patients, FNA was performed and conclusive.

EUS influenced the treatment in 14 (35.0%) patients treated with dCRT or dRT: in 11 (27.5%) it changed the radiation target volumes, in 2 (5.0%) the curability, and in 1 (2.5%) patient FNA was additional. In most of these patients EUS enlarged the radiation target volumes 7 (17.5%), while it was decreased in 4 (10.0%) patients.

### Additional Value of EUS

Additional lymph nodes were detected in 150 (53.8%) patients, whereas less suspicious lymph nodes were found in 32 (11.5%). EUS caused N upstaging in 107 (38.4%) and N downstaging in 31 (11.1%) patients. In 77 (27.6%) patients, EUS found suspicious lymph nodes at other nodal stations than with ^18^F-FDG-PET/CT. In total, 7 (2.5%) patients received a re-EUS examination after primary EUS, because of suspect lymph nodes on the ^18^F-FDG-PET or CT (*n* = 3), an unclear outcome of the FNA (*n* = 3), or an unclear outcome of the EUS itself (*n* = 1).

## Discussion

Accurate staging is of vital importance in the treatment decision-making process and radiotherapy target volume delineation and hence the adjusted correct treatment. The value of EUS after an upfront ^18^F-FDG-PET/CT scan remains matter or debate. Staging with EUS seems required, because it has the highest sensitivity and accuracy in detecting lymph nodes suspicious for metastatic disease, whereas high regional recurrence rates also suggested the need for a more accurate radiotherapy delineation of involved lymph node stations. In clinical practice, not all patients benefit from EUS because of severe stenosis in approximately 20–36%.^11^ The present study is the first to determine the influence of EUS on the given treatment with curative intent. We found that EUS influenced treatment in approximately 29% of the patients. Moreover, EUS was especially important for adequate delineation of radiotherapy target volumes, implicating its importance in locoregional control and treatment of esophageal cancer patients with nCRT.

Several studies previously determined the influence of EUS on treatment decision-making, but not on the given treatment itself. Two studies determined the influence of EUS after CT-alone, with a questionnaire, and found that EUS changed the treatment decision in 24–34% of the patients.^4^,^6^ A study by Van Zoonen determined that although EUS increased the specificity after CT and ultrasound of the neck, the additional value on determining the surgical resectability was limited.^16^ Moreover, Findlay et al. found that the risk of EUS in T2-4a patients on CT outweighed the additional information of EUS on T- and N-stage.^5^ The only study that compared EUS with ^18^F-FDG-PET/CT was the study by Schreurs et al., which found that ^18^F-FDG-PET/CT was the best predictor of curability of the resection.^8^ However, as mentioned, the studies above only determined the influence of EUS on treatment decision making and not on the given treatment itself, whereas detection of metastatic lymph nodes located at some distance of the primary tumor is of fast importance for radiotherapy delineation in the era of nCRT. Currently, the role of EUS in treatment decision making might be limited: In the current treatment paradigm, treatment decision making in T2-4aN0-3M0 esophageal cancer patients is most commonly based on the presence of comorbidities that might not permit nCRT or dCRT. As mentioned, EUS is especially important in patients eligible for nCRT, dCRT, or dRT. Refraining from EUS would have caused inadequate radiotherapy target volumes in approximately 19.6% of the nCRT and 17.5% of the dCRT and dRT patients. In patients eligible for treatment with dCRT and dRT group, this might lead to impaired locoregional control of disease. On the other hand, refraining from EUS would have caused an unnecessary large radiotherapy target volumes in approximately 7% of the patients treated with nCRT and 10% of the dCRT and dRT, which could increase the risk of radiotherapy induced toxicities.

In the future, diffusion-weighted magnetic resonance imaging (DW-MRI) with contrast agents that are taken up by lymph nodes, i.e., Gadofosveset or ultra-small iron particles, might improve the detection of suspicious lymph nodes.^17^ Moreover, in the coming years proton therapy will become more clinically available, which will decrease the radiation associated (cardiopulmonary) toxicities.^18^

An important limitation of this study is its retrospective character, which sometimes impedes the exact anatomical information about suspected lymph node metastases, although all lymph node metastases were scored according to the AJCC system by the researchers. The rate of FNA procedures was rather low in present study (*n* = 58: 20.8%), which may have resulted in false-positive EUS results and probably unnecessary upstaging, because the accuracy of EUS for predicting lymph nodes on echo features in different cancers is approximately 80%.^19^ A large, prospective study with well-defined FNA of suspected lymph nodes might determine the exact role of EUS in up- and downstaging, although accurate EUS with FNA is time-consuming and should be performed in centralized institute because of its relative complexity. In improving the detection of involved regions with potential harmless imaging modalities, the recent use of DW-MRI seems to be of great importance.

In conclusion, our study determined the additional value of EUS on the given treatment and found that EUS was of added value in approximately 29%. EUS seems especially important for radiotherapy target volume delineation of mediastinal and high mediastinal lymph nodes metastases, implicating its importance in staging esophageal cancer patients planned to be treated with curative intended nCRT. Future, prospective studies with current sophisticated imaging would determine the exact place of EUS-FNA in EC staging.
